# Phenotype-specific recombinant haptoglobin polymers co-expressed with C1r-like protein as optimized hemoglobin-binding therapeutics

**DOI:** 10.1186/s12896-018-0424-3

**Published:** 2018-03-15

**Authors:** Christian A. Schaer, Catherine Owczarek, Jeremy W. Deuel, Stefan Schauer, Jin Hyen Baek, Ayla Yalamanoglu, Matthew P. Hardy, Pierre D. Scotney, Peter M. Schmidt, Matthias Pelzing, Peter Soupourmas, Paul W. Buehler, Dominik J. Schaer

**Affiliations:** 1 Division of Internal Medicine, University Hospital Zurich, Ramistrasse 100, CH-8091 Zurich, Switzerland; 2 CSL Limited, Bio21 Institute, Parkville, Australia; 3 Functional Genomics Center Zurich, University of Zurich, Zurich, Switzerland; 4 Division of Blood Components and Devices, Office of Blood Research and Review, Laboratory of Biochemistry and Vascular Biology, FDA, Sliver Spring, MD USA

## Abstract

**Background:**

Preclinical studies have evaluated haptoglobin (Hp) polymers from pooled human plasma as a therapeutic protein to attenuate toxic effects of cell-free hemoglobin (Hb). Proof of concept studies have demonstrated efficacy of Hp in hemolysis associated with transfusion and sickle cell anemia. However, phenotype-specific Hp products might be desirable to exploit phenotype specific activities of Hp 1–1 versus Hp 2–2, offering opportunities for recombinant therapeutics. Prohaptoglobin (proHp) is the primary translation product of the Hp mRNA. ProHp is proteolytically cleaved by complement C1r subcomponent-like protein (C1r-LP) in the endoplasmic reticulum. Two main allelic Hp variants, HP1 and HP2 exist. The larger HP2 is considered to be the ancestor variant of all human Hp alleles and is characterized by an α2-chain, which contains an extra cysteine residue that pairs with additional α-chains generating multimers with molecular weights of 200–900 kDa. The two human HP1 alleles (HP1F and HP1S) differ by a two-amino-acid substitution polymorphism within the α-chain and are derived from HP2 by recurring exon deletions.

**Results:**

In the present study, we describe a process for the production of recombinant phenotype specific Hp polymers in mammalian FS293F cells. This approach demonstrates that efficient expression of mature and fully functional protein products requires co-expression of active C1r-LP. The functional characterization of our proteins, which included monomer/polymer distribution, binding affinities as well as NO-sparing and antioxidant functions, demonstrated that C1r-LP-processed recombinant Hp demonstrates equal protective functions as plasma derived Hp in vitro as well as in animal studies.

**Conclusions:**

We present a recombinant production process for fully functional phenotype-specific Hp therapeutics. The proposed process could accelerate the development of Hb scavengers to treat patients with cell-free Hb associated disease states, such as sickle cell disease and other hemolytic conditions.

**Electronic supplementary material:**

The online version of this article (10.1186/s12896-018-0424-3) contains supplementary material, which is available to authorized users.

## Background

Haptoglobin (Hp) is an abundant plasma protein, which is primarily synthesized in the liver [1]. It is a high affinity scavenger for free hemoglobin (Hb) that is occasionally released from erythrocytes during hemolysis. The complex that is formed between the two proteins (Hb:Hp complex) provides a number of protective activities, which attenuate the toxic impact of free Hb in the kidney, the vasculature and in surrounding tissues accessible to free Hb [2–4]. The protection provided by Hp attenuates two main toxicological consequences of Hb. First, the large molecular size of the Hb:Hp complex prevents extravasation of free Hb [5]. This mechanism protects renal function and preserves vascular nitric oxide (NO) homeostasis by limiting access of free Hb into the vascular wall [6]. Secondly, Hb:Hp complex formation stabilizes the structure of the Hb molecule in a way that limits transfer of heme from its globin chains to proteins and reactive lipids [7–10]. These mechanisms are largely responsible for Hp’s anti-oxidative function during hemolysis.

Cell free Hb is increasingly recognized as an amplifier of disease in hemolytic anemias [11, 12]. While endogenous Hp could principally provide significant protection against free Hb toxicity, it is rapidly consumed and depleted during more pronounced acute or prolonged hemolysis [13]. Replacement of Hp has therefore being considered as a therapeutic modality demonstrating preclinical proof-of-concept in vitro and in animal models of hemolysis [14–18]. So far, preclinical studies have evaluated Hp purified from pooled human plasma fractions. However, this approach may present relevant limitations in clinical translation, including; (1) the mixture of different Hp phenotypes (1–1, 2–1 and 2–2) may trigger neutralizing antibody responses in some patients during prolonged replacement therapy, (2) differing phenotypes may afford differing efficacy and (3) phenotypic forms may demonstrate different pharmacokinetics. Considering the potential limitations of plasma derived Hp, recombinant protein production may offer a relevant strategy for production. Additionally, recombinant protein-production strategies may also generate therapeutics with enhanced functionality, bioavailability and pharmacokinetics.

**Fig. 1:**
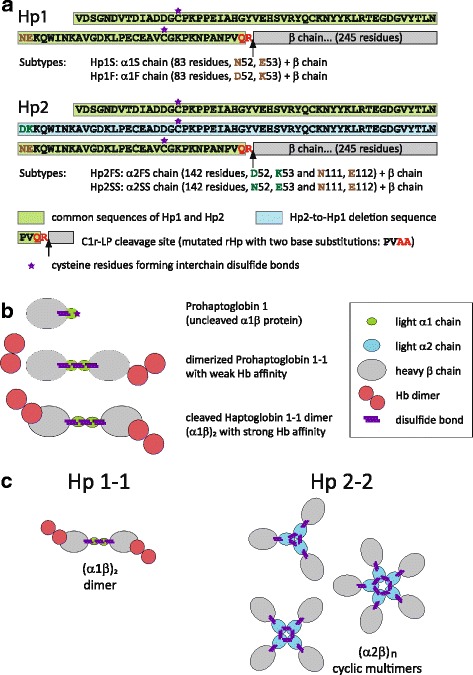
Haptoglobin structure. **a** Schematic representation of Hp1 and Hp2. The common α-chain sequence of Hp1 and Hp2 is green. The blue colored amino acid sequence within the α-chain of Hp2 determines the distinct molecular phenotypes. This sequence corresponds to exons 3 and 4 of the ancestral HP2 allele and was deleted due to non-allelic homologous recombination during the structural evolution of HP2 to HP1. The cysteine residues that participate in protein multimerization are marked with a purple star. Both Hp1 and Hp2 further segregate into subtypes S and F or FS and SS, respectively, based onto two base mutations at positions 52 and 53 or 111 and 112. The C1r-LP cleavage sites with the red colored two base substitution sites in the mutated rHp are marked with black arrows. **b** Dimerization and proteolytic C1r-LP cleavage of pro-Hp 1 to Hp 1–1 dimer. **c** Protein quaternary structure of the Hp 1–1 homo-dimer with one inter-α-chain disulfide bond and three variants of Hp 2–2 cyclic homo-multimers with two inter-α-disulfide bonds

Prohaptoglobin (proHp) is the primary translation product of the Hp mRNA. In the endoplasmic reticulum proHp dimerizes via disulfide bond formation and is proteolytically cleaved by the protease complement C1r subcomponent-like protein (C1r-LP) [19, 20]. As a result, Hp exists in most mammals as a dimeric protein of 150 kDa consisting of two light α-chains and two heavy β-chains that are linked by a single disulfide bond (S-S) between the two α-chains, yielding Hp 1–1 **(**Fig. 1) [21]. Interactions with Hb and the clearance receptor CD163 are mediated by the larger and more accessible β-chain [21]. In humans there exist two main allelic Hp variants, HP1 and HP2. According to more recent genetic analyses the larger HP2 is now considered to be the ancestor variant of all human Hp alleles [22]. The HP2 variant is characterized by an α2-chain, which contains an extra cysteine residue that pairs with an additional α-chain leading to the formation of a heterogeneous mixture of multimers with molecular weights of 200–900 kDa (Hp 2–2). The two human HP1 alleles (HP1F and HP1S), which differ by a minor two-amino-acid substitution polymorphism within the α-chain, derived from HP2 by recurring exon deletions. There exist no known functional differences between these two HP1 variants.

In the present study, we describe a process for the production of recombinant phenotype specific Hp proteins. We demonstrate that efficient expression of mature and fully functional Hp in mammalian cells requires co-expression of active C1r-LP.

## Methods

### Materials

Throughout this article, molar quantities of Hb relate to total heme, which is equivalent to the single chain subunits of Hb (α- or β-chain). For the scavenger proteins (Hpx and Hp), we considered one mole as the binding capacity equivalent for one mole of heme, considering that one Hp-αβ-subunit (≈ 42 kDa) can bind two heme molecules (corresponding to one αβ-dimer). Hb concentration was measured by spectrophotometry using a molar extinction coefficient of 524.3 M^− 1^ cm^− 1^ at an absorbance wavelength of 414 nm (for oxyHb). MetHb or ferric Hb (Fe^3+^), respectively, was generated by incubating oxyHb (Fe^2+^) with a five times molar excess of K_3_[Fe (CN)_6_] in PBS, pH 7.45 at room temperature for 10 min followed by removal of K_3_[Fe (CN)_6_] by size exclusion chromatography on a PD-10 column (GE Healthcare). Ferric Hb concentration and complete oxidation to ferric iron (Fe^3+^) within Hb where then determined by spectrophotometry. Human plasma derived Hp (predominantly phenotype 2–2) and Hpx were provided by CSL Behring (Bern, Switzerland). Reconstituted lipoprotein (rLP; CSL-111) was obtained from CSL Behring (Switzerland). rLP was reconstituted in PBS to a final stock concentration of 40 g/L and immediately aliquoted and stored at − 80 °C until use. For rLP, all given concentrations are relative to the apoA1-lipoprotein concentration. The lipoprotein is composed of human plasma derived apoA1 lipoprotein and lecithin with phosphatidylcholine as the principal lipid component.

### Generation of cDNA expression plasmids

In humans Hp exists in two allelic forms; Hp1 and Hp2 (Hp2FS, longer isoform, Genbank Accession number NP_005134.1), which differ only in their respective α-chains. The Hp1 allele can be further subdivided into Hp1F (Genbank Accession number NP_001305067.1, Asp52, Lys53) and Hp1S (Genbank Accession number NP_001119574.1 Asn52, Glu53). Human Haptoglobin cDNAs were codon-optimized for human expression and synthesized by Geneart (Invitrogen™, Thermo Fisher Scientific) each with a Kozak consensus sequence (GCCACC) immediately upstream of the initiating methionine (+ 1). C-terminal 8× Histidine-tags were fused in-frame using standard PCR-based mutagenesis techniques. Once each cDNA was completed, it was digested with *Nhe*I and *Xho*I and ligated into pcDNA3.1 (Invitrogen™, Thermo Fisher Scientific). Transient transfections of expression plasmids using FS293F cells were performed using 293fectin™ transfection reagent (Invitrogen™, Thermo Fisher Scientific). To generate completely processed Hp1F, Hp1S and Hp2FS proteins cDNAs encoding these proteins were co-transfected with a cDNA (comprising 5% of the total DNA in the transfection reaction) encoding human complement C1r subcomponent-like protein (C1r-LP, Genbank Accession number NP_057630), which mediates the proteolytic cleavage of haptoglobin in the endoplasmic reticulum, with a FLAG (DYKDDDDK) epitope tag encoded at its C-terminus. Uncleaved Hp, also known as Zonulin was generated by alanine mutagenesis of the C1r-LP cleavage sites in Human Hp1S at Q101 and R102, and Human Hp2FS at Q160 and R161.

### Purification of recombinant Haptoglobin variants

Purification of recombinant Hp variants was performed by two-step chromatography using an ÄKTA Express system (GE Healthcare Bio-Sciences, PA, USA) equipped with 1 mL HisTrap columns (GE Healthcare Bio-Sciences, PA, USA) followed by size exclusion chromatography (SEC) on a 16/60 Superdex 200 column (GE Healthcare Bio-Sciences, PA, USA) equilibrated with PBS. Hp-containing fractions were pooled and concentrated using Amicon Ultra-15 centrifugal filter units (Merck-Millipore, MS, USA) according to the manufacturer’s protocol. The concentration of purified recombinant proteins was measured by OD280 on a Trinean DropSense96 system (Trinean, Gentbrugge, Belgium) and purity of the concentrates was verified by SDS-PAGE (NuPAGE system, Thermo Fisher Scientific, MA, USA).

### Surface plasmon resonance (SPR) and size exclusion chromatography

Surface plasmon resonance (SPR) measurements were performed on a ProteOn XPR36 instrument (Bio-Rad, Reinach, Switzerland). Hp was immobilized on a ProteOn Sensor Chip GLM using standard amine coupling chemistry. The running buffer was 150 mM NaCl, 10 mM HEPES (pH 7.6), 3 mM EDTA and 0.05% (*v*/v) polysorbate 20 at + 25 °C. Sensograms were interspot referenced and kinetic analysis performed by globally fitting the corrected data to a Langmuir 1:1 binding model using the manufacturers software (ProteOn Manager Software 3.1.0.6). For size-exclusion chromatography (HPLC) mixture of purified Hb and Hp variants or plasma samples were separated on an analytical BioSep-SEC-S3000 (600 × 7.5 mm) column (Phenomenex, Torrance, CA) attached to a Waters 2535 quatrinary gradient module, Waters 2998 photodiode array multi-wavelength detector, and controlled using Empower Pro software (Waters Corp., Milford, MA). The mobile phase was 50 mM Potassium Phosphate, pH 7.4. The absorption wavelengths were set at λ = 280 nm, λ = 405 and 413 nm to detect heme in protein.

### Vascular function studies

Vascular function studies were performed as described elsewhere [5]. In short, rings (approx. 7 mm in length) of porcine left anterior descending coronary arteries (LAD) were prepared and mounted under 40 mN resting tension, via two stainless steel hooks, in a 10 mL organ bath containing prewarmed (37 °C) and aerated (95% O2 and 5% CO2) Krebs-Henseleit Solution (118 mM NaCl, 4.7 mM KCl, 1.2 mM MgSO4, 2.5 mM CaCl2, 1.2 mM KH2PO4, 25 mM NaHCO3, 11 mM glucose, pH adjusted to 7.4). Measurement of isometric tension via a force displacement transducer (Biegestab K30, Hugo Sachs Elektronik – Harvard Apparatus GmbH, 29,232, March, Germany) was digitally recorded. As precontracting agent, prostaglandin F2α (PGF2α; Sigma, Buchs, Switzerland) was used at a 10 μM concentration and nitric oxide synthase was irreversible inhibited by L-N5-(1-Iminoethyl)-ornithine hydrochloride (L-NIO; Sigma) at a 10 μM concentration. 30 nM of the extracellular NO donor MAHMA-NONOate (ENZO Life Sciences, Lausen, Switzerland) were injected into the immersion buffer to induce a transient and short-lived NO-mediated dilatory response of the PGF2α precontracted porcine coronary artery segments. Hb and all other compounds were used in concentrations indicated in the figure legends. Hp was always used in 1.2 M excess relative to Hb. The recorded vascular function responses were normalized relative to the 40 mN baseline tension (= 100%) and the maximal PGF2α contraction (= 0%). Responses in the plots are therefore indicated as [% PGF2α]. Normalized segments were subsequently plotted (mean ± SEM) and analyzed in GraphPad Prism Software Version 5.0.

### Animal experiments

All animal studies were approved by the FDA Institutional Animal Care and Use Committee, with all experimental procedures performed in adherence to the NIH guidelines on the use of experimental animals. Male Hartley Guinea Pigs weighing 300–400 g were obtained from Charles Rivers Laboratories (Wilmington, MA) and acclimated upon arrival at the FDA animal care facility (The Federal Research facility at White Oak, Silver Spring, MD). For Hb and Hp administration and systemic arterial blood pressure recordings indwelling, fluid-filled catheters were placed into the jugular vein and carotid artery, respectively. Animals were dosed with 25 mg (~ 1552 mmol) of human Hb in saline (0.3 mL total volume) to establish a maximum MAP response. At 5 min post Hb dosing rHp 1–1 or rHp 2–2 was administered at a Hb equimolar dose via an intravenous (i.v., jugular) infusion to guinea pigs (*n* = 4) to evaluate the blood pressure response post Hb and rHp dosing and to perform blood collection for determination of Hb complexation with rHps in plasma. Systemic blood pressure and heart rate was measured from the indwelling carotid catheter using a Gould P23XL pressure transducer (Becton Dickinson, Singapore) connected to a Transonic Powerlab 4/30 (Transonic Systems, Ithaca, New York). Blood pressure and heart rate were measured at baseline (30 min), after Hb infusion (5 min) and for 30 min post rHp 1–1 or rHp 2–2. Plasma (0.1 mL) was collected at baseline, 5 min post Hb infusion and 5, 30 min and 60 min post rHp dosing to establish the percent of Hb bound to Hp.

### Hemopexin capture assay

Heme release from metHb (Fe^3+^) was measured using a hemopexin capture assay that was described earlier [23]. The transfer of heme to Hpx was measured as gradual disappearance of the characteristic metHb (Fe^3+^) UV-VIS spectrum and an increase of the spectrum that is specific for the heme-Hpx complex. Reactions were measured with a Cary 60 UV-VIS spectrophotometer (Agilent Technologies, Switzerland). Experiments were conducted in 1 mL semi-micro cuvettes at 37 °C and absorbance was measured over time at every 2 nm between 350 and 650 nm with a 0.025 s integration time per wavelength and time-point. The concentrations over time were calculated by multicomponent spectral deconvolution against standard extinction curves of HbFe^3+^ and heme-Hpx using a non-negative least squares algorithm.

### Lipoprotein peroxidation

Experiments were conducted in 1 mL semi-micro cuvettes at 37 °C using a Cary 60 spectrophotometer (Agilent, Switzerland). Absorbance was measured over time at every 2 nm between 350 to 650 nm with a 0.025 s integration time per wavelength and timepoint. Spectra were deconvoluted against standard extinction curves of oxyHb (Fe^2+^), deoxyHb (Fe^2+^) and metHb (Fe^3+^) using a non-negative least squares algorithm. The time to oxygen depletion was calculated as the minimum of the first derivative of the oxyHb concentration over time [7].

### Cell culture experiments

Human umbilical vein endothelial cells (HUVEC) were obtained from Lonza (Switzerland) and cultured in EGM medium (Lonza) under standard conditions. All experiments were conducted in complete EGM without ascorbic acid with cells forming a confluent monolayer.

### Fluorescence microscopy

HUVEC cells were grown on sterile, round 12-mm glass coverslips (Hecht-Assistent, Sondheim, Germany) coated with rat-tail collagen (BD Biosciences, San Jose, CA, USA) in 24-cluster wells. After treatment, cells were washed with PBS, pH 7.4 (Sigma Chemical Co.), fixed with 2.5% paraformaldehyde in PBS for 15 min and permeabilized with 0.1% Triton X-100 (Sigma Chemical Co.) in PBS for 5 min at room temperature. After washing, nonspecific binding sites were blocked with 10% goat serum and 1% bovine serum albumin (Sigma Chemical Co.) in PBS for 1 h at room temperature. Adherens junction protein staining was performed with a polyclonal rabbit anti-human β-catenin antibody (Cell Signaling Technology, Danvers, MA, USA) overnight at 4 °C at a dilution of 1: 50 in PBS with 1% goat serum and 0.1% bovine serum albumin. As a secondary antibody a Cy3-labeled polyclonal sheep anti-rabbit antibody (IgG fraction; Abcam, Cambridge, UK) was used at a dilution of 1:50. Nuclear counterstaining was performed with 10 μg/mL 4,6-diamidino-2-phenylindole (DAPI; Sigma Chemical Co.). After washing, coverslips were mounted with ProLong Gold antifade reagent-mounting medium (Molecular Probes, Invitrogen, Eugene, OR, USA) and visualized using a Zeiss Observer.Z1 with a Colibri.2 and ApoTome.2 system (Carl Zeiss AG, Feldbach, Switzerland) at a 400 times original optical magnification.

### Electrical cell impedance sensing (ECIS)

Confluent HUVEC cells grown in 10-cm tissue culture plates were trypsinized and plated into 8W10E+ ECIS plates filled with 400 μL medium per well. After 24 h, the medium was changed to 300 μL per well. After stabilization of the ECIS signal, 100 μL of the 4× reaction mixture was added directly to the cells while recording the electrical cell impedance at 4 kHz on an ECIS Z Theta instrument (ibidi GmbH, Planegg/Martinsried, Germany) with ECIS software version 1.2.52. After signal normalization (mean pre-experimental signal), the amount of time required to lose endothelial monolayer integrity was calculated by finding the minimum in the first derivative of the measured resistance over time.

## Results

### Co-expression of C1r-LP is required for efficient production of mature haptoglobin in mammalian cells

**Fig. 2:**
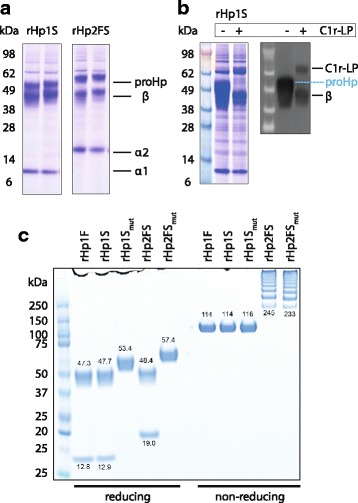
Haptoglobin and C1r-LP co-expression in FS293F cells. **a** Coomassie stained reducing SDS-PAGE of rHp1S and rHp2FS produced in transfected FS293F cells. Hp α-chain appears at 12 or 19 kDa and Hp β-chain appears at 47 kDa. Additionally uncleaved proHp1 and proHp2 appear at their expected size of 53 or 57 kDa. **b** With co-expression of C1r-LP (+) all proHp is efficiently cleaved into its subunits. C1r-LP appears at 68 kDa. The right image shows an anti-8His Western blot, which shows uncleaved proHp in the absence of C1r-LP and smaller Hp β-chain as well as the His-tagged protease in the presence of C1r-LP co-expression. **c** The Coomassie stained gel of reduced protein samples (left side) illustrates that the cleavage-site mutated (mut) Hp variants remain uncleaved and appear as single proHp bands at their expected size. With non-reduced protein samples polymers of Hp 1–1 and Hp 2–2 can be resolved in wild-type as well as in mutated variants

Overexpression of recombinant Hp in FS293F cells in the absence of sufficient C1r-LP is not optimal because it results in a significant amount of uncleaved proHp. Figure 2a shows a Coomassie-stained reducing SDS-PAGE of unpurified recombinant Hp produced in FS293F cells. The left image shows secreted Hp 1–1 produced by rHP1S transfected cells. The right image shows rHp 2–2 produced by rHP2FS transfected cells. In addition to some cleaved Hp α- (12 and 19 kDa) and β- (47 kDa) chains a larger uncleaved protein corresponding to uncleaved proHp (53 and 57 kDa) is present. To overcome the problem of inefficient proHp processing in FS293F cells we have co-expressed Hp with the cleavage protease C1r-LP, similar to the C1r-LP coexpression in COS-1 cells in an earlier study [20]. Figure 2b shows a Coomassie stained gel (left image) and an anti-8His Western blot (right image) of secreted Hp1S, which was produced with (right lane) and without (left lane) C1r-LP co-expression. With C1r-LP co-expression proHp is entirely cleaved resulting in mature Hp consisting of α- and β-chains. Since for this experiment C1r-LP was expressed as an 8-His tagged protein the protease can also be visualized at 68 kDa. The recombinant Hps produced by this strategy are glycosylated, although with a different glycan pattern compared to plasma derived Hp as determined by HPLC-ESI-MS. The glycan analysis data are shown in the Additional file 1.

C1r-LP cleaves proHp after arginine R102 (P-V-Q-**R** ↓) in Hp1F and Hp1S or after R161 in Hp2FS (Fig. 1a**)**. To understand the functional importance of Hp maturation, we have produced recombinant Hp variants with amino-acid substitutions within the predicted cleavage site of Hp1S (Q101A, R102A) and Hp2FS (Q160A, R161A). The Coomassie-stained gel shown in Fig. 2c confirms that the two cleavage-site mutated Hp variants remain uncleaved as indicated by the absence of the α-chain subunit under reducing conditions. However, subunit crosslinking and multimerization appear to be unaffected in uncleaved Hp. Under non-reducing conditions wild-type and mutant Hp variants have an identical appearance as a single (α1β)_2_ dimer band of 114–116 kDa in case of Hp 1–1 and a series of (α2β)_n_ multimers in case of Hp 2–2.

### C1r-LP processing of Hp is mandatory for high affinity Hb binding and irreversible complex formation

**Fig. 3:**
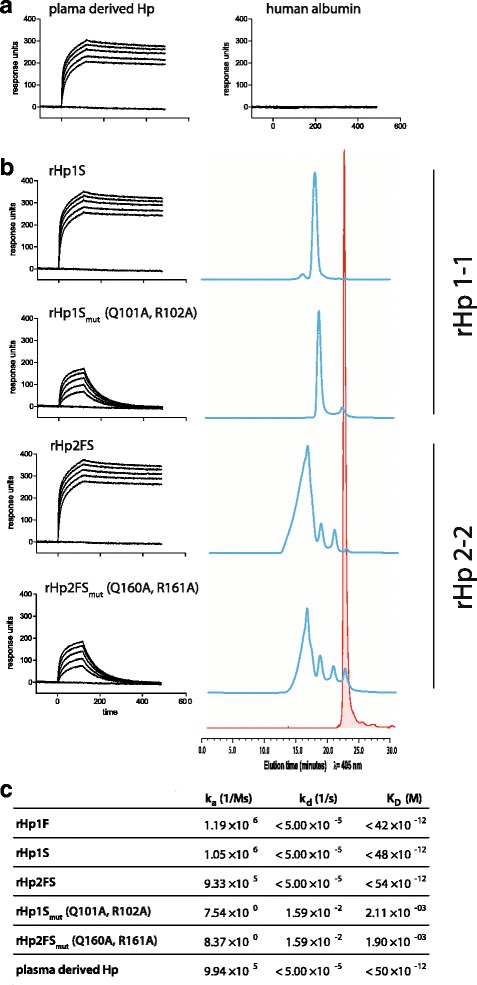
Hb-binding activity of plasma derived and recombinant Hp. **a** Human plasma derived Hp (left) or albumin (right) were immobilized on the SPR-Chip and tested for Hb-binding activity by surface plasmon resonance (SPR). The lines represent the association and dissociation signal curves of a range of Hb concentrations in the fluid-phase (0, 312.5, 625, 1250, 2500 and 5000 nM). **b** SPR analyses of rHp variants with (mut) and without cleavage-site mutations (left panels) with the corresponding size exclusion HPLC chromatographs (right panels). The blue lines represent the signals of the Hb + rHp mixtures, the red line shows the signal of Hb alone. All size exclusion HPLC traces are scaled identically to fit to the red Hb peak chromatogram. **c** Association (k_a_), dissociation (k_d_) and equilibrium (K_D_) constants for the SPR experiments

We have measured the Hb-binding kinetics of rHp by surface plasmon resonance (SPR). Figure 3 shows association and dissociation signal curves of a concentration range of Hb run against immobilized Hp. Immobilized plasma derived Hp of mixed phenotype and human albumin were used as positive and negative controls **(**Fig. 3a**)**. Plasma derived Hp and the C1r-LP processed Hps (rHp1S and rHp2FS) show an identical Hb binding pattern with rapid association and hardly detectable dissociation, which is typical for the irreversible complex formation between the two proteins. In contrast, the cleavage-site mutated Hp variants (rHp1S_mut_ and rHp2FS_mut_) show a lower affinity reversible Hb-binding, which was nevertheless significant compared to the completely absent binding of Hb to immobilized albumin. Rates of complex formation (k_a_) and dissociation (k_d_) are listed in Fig. 3c. The equilibrium constant K_D_ was calculated from the two kinetic constants through the defining relation K_D_ = k_d_/k_a_.

The SPR derived binding properties are supported by size-exclusion chromatography analyses of equimolar mixtures of Hb with the different rHp variants (Fig. 3b). Wild-type and mutant rHp do both resolve Hb:Hp complexes as the main fraction, indicating that even uncleaved rHp forms low-affinity complexes with Hb. However with rHp1S_mut_ and rHp2FS_mut_ there appears also a significant peak of free Hb in addition to the larger Hb:Hp complex fraction. This indicates that the low affinity reversible Hb binding of these proteins results in an equilibrium of complex and unbound Hp and Hb, respectively.

### Recombinant haptoglobin preserves vascular nitric oxide signaling in vitro and in vivo

**Fig. 4:**
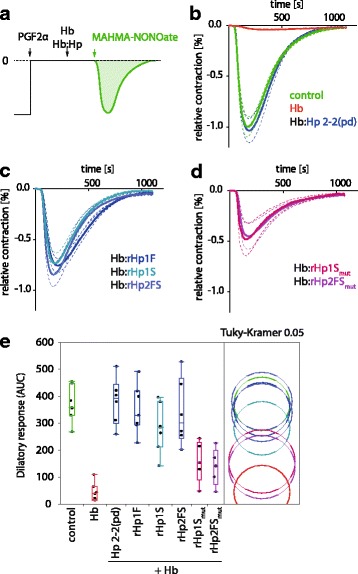
Vascular nitric oxide (NO) signaling. **a** Schematic illustration of the experimental setup. Porcine coronary artery segments were mounted under 40 mN resting tension in an organ bath and pre-contracted with PGF2α (= baseline). NO-mediated dilatory responses were recorded after injection of the NO donor MAHMA-NONOate (30 nM) into the immersion buffer. **b** Hb (32 μM) but not Hb complexes with predominantly human plasma derived (pd) Hp 2–2 blunted the dilatory response. **c** and **d** Wild-type rHp variants preserved vascular NO-signaling comparable to plasma derived Hp. In contrast, intermediate dilatory responses were observed in the presence Hb mixed with cleavage site mutated rHp variants. The traces show mean +/− SEM of at least 6 averaged responses recorded in three independent experiments. **e** ANOVA statistics of the AUCs of the dilatory responses recorded in the different experiments. Non-overlapping circles indicate significantly different responses at *p* < 0.05 (Tukey-Kramer post-test)

Preservation of arterial nitric oxide (NO) signaling is a key function of Hp, which directly relates to the protein’s capacity to sequester Hb in a Hb:Hp complex. The large complex cannot extravasate and does therefore not access subendothelial vascular sites of NO production and signaling. To explore the functional activity of recombinant Hp proteins and, more specifically, the requirement of Hp subunit cleavage in vascular protection, we measured how the different rHp proteins could revert the disruptive activity of cell-free Hb on NO mediated arterial relaxation. Rings of porcine coronary arteries show a transient dilatory response after addition of the short-lived NO donor MAHMA-NONOate (Fig. 4a and b, green line). This dilatory response was completely blunted in the presence of cell-free Hb (32 μM) (Fig. 4b, red line). Plasma derived Hp completely restored the dilatory activity of NO when present in a ≥ 1:1 M concentration relative to Hb (Fig. 4b, blue line). The recombinant Hp rHp1F, rHp1S and rHp2FS demonstrated protective effects that were comparable to the effects of plasma-derived Hp (Fig. 4c and e). In contrast, the cleavage-site mutated Hp variants rHp1S_mut_ and rHp2FS_mut_ only partially restored arterial NO responses (Fig. 4d and e), indicating that the irreversible high-affinity Hb binding is critical for the NO preserving function of Hp. A summary and statistical comparison of all rHp effects expressed as area under the curve (AUC) of the dilatory responses is shown in Fig. 4e.

**Fig. 5:**
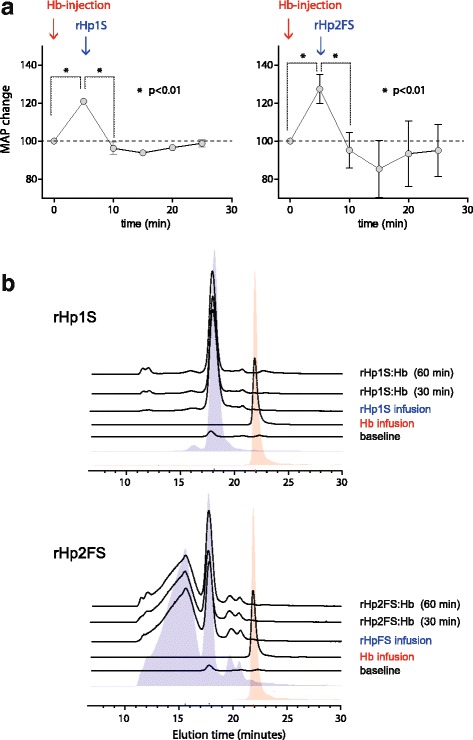
Blood pressure control and in vivo Hb:Hp complex formation. **a** Guinea pigs (*n* = 4 per group) were infused with 25 mg of Hb at time 0 min. At *t* = 5 min, when mean arterial blood pressure (MAP) reached a maximum increase rHp (25 mg) was injected. Separate groups were treated with rHp1S (left) and rHp2FS (right). Data show mean ± SD. Values were compared with an ANOVA for repeated measures and a Tukey post-hoc test correction for multiple comparisons. **b** Plasma samples collected at baseline, 5 min post Hb infusion and 5, 30 and 60 min post rHp injection were analyzed by HPLC size-exclusion chromatography. The red area indicates the elution profile of a cell-free Hb standard, the blue area indicates the elution profile of the respective Hb:Hp complexes with rHp1S and rHp2FS

In vivo the protection of NO signaling by Hp translates into a hemodynamic stabilization during Hb infusion. When guinea pigs were infused with a bolus of 25 mg oxyHb a rapid increase in mean arterial blood pressure (MAP) could be observed (Fig. 5a), which was associated with the appearance of cell-free Hb in plasma (Fig. 5b). Injection of 25 mg rHp1S or rHp2FS at the time of peak blood pressure immediately eliminated free Hb in plasma to undetectable levels, while blood pressure declined to baseline. After injection of rHp all the Hb absorbance signal could be detected within the large molecular size Hb:Hp complex fractions, which appeared as a monomeric signal after rHp1S treatment or as a multimeric signal after rHp2FS treatment. When saline or albumin were infused instead of rHp blood pressure remained around peak levels for up to 1 hour, additionally, rHp 1–1 and rHp 2–2 administered in absence of Hb showed no effect on blood pressure or heart rate after repeat dosing (data not shown).

### rHp prevents heme release and lipid peroxidation

**Fig. 6:**
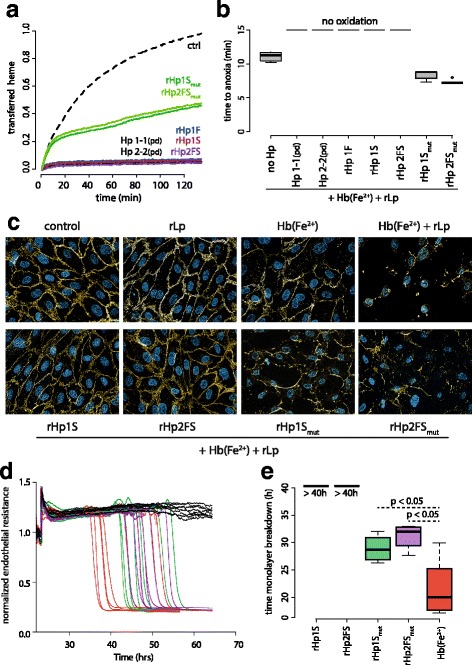
rHp prevents heme release and lipid peroxidation. **a** Heme transfer from metHb to hemopexin was measured by spectrophotometry in a reaction mixture of 10 μM metHb(Fe^3+^) and 10 μM hemopexin. Plasma derived (pd) and recombinant wild-type Hp at 10 μM concentration completely blocked the reaction while only partial inhibition was observed with the cleavage site mutated rHps (green lines). **b** Lipoprotein peroxidation was measured by spectrophotometry as a function of oxyHb deoxygenation in a reaction mixture of 10 μM oxyHb(Fe^2+^) with 0.5 g/L rLP. The graph shows the time to anoxia. Peroxidation was completely blocked for > 6 h by the plasma derived and recombinant wild-type Hps, whereas lipid oxidation was not delayed compared to free Hb by the cleavage-site mutated rHp variants rHp1S_mut_ and rHp2FS_mut_. **c** The identical lipid peroxidation reaction of rLP (0.5 g/L) and oxyHb (10 μM) as described in (**b**) was performed in cultures of HUVEC cells. Toxicity of lipid peroxidation products was monitored by fluorescence microscopy of β-catenin (yellow) and nuclear morphology (DAPI, blue) at 24 h after initiation of the reaction (original optical magnification 400×). Identical experiments as in (**c**) were performed on HUVEC monolayers monitored in an ECIS instrument. **d** Shows original electrical monolayer resistance data over time of biologic replicates. The colors represent the different treatments as indicated in (**e**). **e** “Time to monolayer breakdown” was calculated as a derivative from the electrical resistance data shown in (**d**). Data indicates mean ± SD of 6 biologic replicates

Heme release from metHb (Fe^3+^) is a surrogate marker of the oxidant capacity of Hb and can be measured as heme transfer in a reaction mixture of metHb (Fe^3+^) with the high affinity heme-scavenger protein hemopexin (Hpx), which exerts a specific absorption spectrum as heme-hemopexin complex. Plasma derived Hp as well as rHp1F, rHp1S and rHp2FS completely blocked heme-transfer. In contrast the cleavage site mutated rHp variants rHp1S_mut_ and rHp2FS_mut_ only partially impaired transfer of heme from metHb (Fe^3+^) to hemopexin (Fig. 6a).

One potential pathway of oxidative Hb toxicity relates to the oxidation of lipoproteins by cell-free Hb, which may result in the generation of toxic lipid oxidation products. We have recently described a kinetic assay which measures oxygen consumption as a surrogate marker of lipid peroxidation in the reaction of oxyHb with a reconstituted lipoprotein (rLP). In this assay, spectrophotometric detection of oxygen desaturation of Hb (oxyHb (Fe^2+^) ➔ deoxyHb (Fe^2+^)) is used as a reaction-intrinsic sensor for oxygen depletion in an air-tight system. Figure 6b shows that oxygen becomes depleted around 10 min after start of a reaction with 10 μM of oxyHb and 0.5 g/L rLP. In contrast in the presence of equimolar concentrations of plasma derived Hp or rHp1F, rHp1S or rHp2FS no oxygen depletion could be observed even after > 6 h, indicating that these proteins fully interrupt lipid peroxidation by Hp. In contrast, cleavage-site mutated rHp variants did not provide any significant (anti-oxidant) protection in this assay.

Biologic toxicity resulting from cell-free Hb triggered lipid peroxidation can also be measured in an in vitro cell culture assay using the disruption of endothelial monolayer integrity as an endpoint. Fluorescence microscopy analyses of the distribution of the junctional protein β-catenin indicate that the in-situ reaction of 10 μM Hb (Fe^2+^) and 0.5 g/L rLP caused disruption of monolayer integrity within 24 h after addition of the reactants to the cell culture medium (Fig. 6c). In contrast, when recombinant rHp1S or rHp2FS were also added at equimolar concentration to the reaction the endpoint was not reached. Cleavage-site mutated rHp1S_mut_ and rHp2FS_mut_ did not visually alter β-catenin distribution compared to the experiments performed in the absence of Hp. Figure 6d shows raw ECIS transendothelial resistance measurement traces of individual replicates of HUVEC monolayers incubated with rLP alone (black lines), rLP + Hb (Fe^2+^) (red lines) or rLP + Hb + different Hp variants (green, purple and black lines). Figure 6e shows the pooled first derivative data of these traces, which indicate an estimate for the time-point of monolayer breakdown. The data confirm the morphologic data demonstrating complete monolayer protection with the wild-type rHp1s and rHp2FS (black) and minimal, though statistically significant, protection with cleavage site mutated Hps (green and purple).

## Discussion

Haptoglobin is a candidate therapeutic protein for diseases that are caused or amplified by the pathophysiological effects of cell-free Hb [11]. Among the potential indications for Hp are the hereditary hemolytic anemias such as sickle cell disease, procedure-related hemolysis during open heart surgery or subarachnoid hemorrhage. So far, human pooled plasma has been used as the source for experimental Hp products and one plasma-derived Hp product is approved and marketed in Japan since 1984. While the use of these products appears to be safe and effective in several preclinical disease models and in patients with acute hemolysis, recombinant production processes for Hp may offer several advantages, which may enhance the properties of plasma derived Hp in therapeutic applications.

Two exons of the Hp exhibit copy number variations that affect Hp protein structure and multimerization patterns. The distribution of the different phenotypes varies across populations worldwide depending upon racial origin. The allelic prevalence of HP1 for example approaches about 0.07 in parts of south Asia with increasing occurrence north- and westwards up to 0.7 in parts of west Africa and South America. This results in a phenotype distribution of dimeric Hp 1–1, hetero-multimeric Hp 2–1 and homo-multimeric Hp 2–2 of roughly 0.1, 0.4 and 0.5 in Asia, 0.15, 0.5 and 0.35 in Europe/North America, 0.3, 0.35 and 0.15 in Africa (0.2 anhaptoglobinemic) and 0.5, 0.4 and 0.1 in South America [24]. Pooled human plasma derived Hp is therefore largely composed of multimeric protein species. Although the antioxidant and vascular NO-sparing functions of Hp 1–1 and Hp 2–2 appear to be comparable if examined in vitro or in animal studies investigating short-term therapeutic end-points [23], several epidemiologic studies suggest that Hp 2–2 may be associated with inferior cardiovascular outcomes [25–27]. A large genome-wide association study (GWAS) in more than 20′000 individuals found that the Hp exonic deletions characteristic of Hp 1–1 are associated with lower plasma total and LDL cholesterol concentrations [22, 28]. The observation that lower cholesterol was found in individuals with both genotypes (HP1F and HP1S), which were present in considerably different SNP haplotypes, suggest that cholesterol levels are related to structural Hp variation rather than nearby polymorphisms in unrelated genes. It is unclear so far whether the beneficial effects of Hp 1–1 (or the adverse effects of Hp 2–2) on cholesterol metabolism could be transferred by phenotype specific therapy. However, the concept should be considered in anticipated clinical trials exploring the effects of chronic Hp supplementation in patients with hemolysis. The availability of recombinant phenotype specific therapeutics may support such trials. Recombinant technology may also allow targeted modification of the Hp protein to enhance functionality or pharmacokinetic properties such as bioavailability and clearance of the Hb:rHp complexes by the macrophage CD163 scavenger receptor system.

Hp is a large glycoprotein that undergoes complex posttranslational modifications, such as disulfide-bridge multimerization and cleavage of proHp [19, 20]. The functional relevance of this modification, which ultimately produces a hetero-multimeric protein composed of α- and β-subunits remained unclear. In our studies, we established a production process, which is based on co-expression of proHp with its cleavage protease C1r-Lp. We observed that co-expression of the two proteins dramatically enhanced production efficiency and almost entirely eliminated the generation of immature protein. This effect was equally effective for all tested genetic Hp variants, indicating that both rHp 1–1 and rHp 2–2 could be produced using this approach. rHp 1–1 and rHp 2–2 produced by this process appeared fully functional, when compared with plasma derived human Hp. rHp formed irreversible complexes with cell-free Hb in vitro and in vivo, it preserved vascular NO signaling in the presence of Hb in porcine coronary arteries and abrogated the acute hypertensive effect of cell-free Hb in rats. rHp was also equal to plasma-derived Hp in preventing heme-release from metHb and in blocking lipoprotein oxidation by oxyHb.

We have also explored the functional demand for proHp processing by C1r-LP. For these experiments, we expressed variants of Hp1S and Hp2FS with targeted mutations in their respective C1r-LP cleavage site. The introduced amino acid substitutions completely prevented segregation of α- and β-chains, yielding unprocessed proHp for functional studies. Compared to the mature protein, unprocessed Hp demonstrated reversible Hb binding and incomplete complex formation with a considerable fraction of free Hb even when excess rHp was present. Accordingly, unprocessed Hp only partially protected vascular NO-signaling and it was less effective in preventing heme release from metHb compared to plasma-derived Hp and mature rHp. Interestingly, the mutated Hp variants were weak inhibitors of Hb triggered lipoprotein oxidation, suggesting that the irreversible high affinity complex formation between Hb and Hp are an absolute requirement for its antioxidant function and it might, in part, be this function of Hp which has driven the evolution of one of strongest protein-protein interactions found in nature. While our results establish that high affinity and irreversible binding between Hp and Hb are an absolute requirement for Hp’s protective activity against Hb toxicity, our studies cannot definitely define the role of C1r-LP processing in the “functional maturation” of the protein. The peptide chain mutations that we introduced to prevent proHp cleavage could theoretically reduce Hb-binding affinity through introduction of additional conformational deteriorations in the protein. In fact, other authors found that recombinant, mainly uncleaved Hp expressed in COS cells demonstrated considerable Hb binding activity [29].

## Conclusion

We provide evidence that the high affinity, irreversible complex formation between Hp and Hb is essential for Hp’s functions in the detoxification of free Hb. The proposed production process of fully functional and phenotype-specific recombinant Hp could accelerate the development of Hb scavengers to treat patients with cell-free Hb associated disease states, such as sickle cell disease and other hemolytic conditions.

## Additional file

Additional file 1: **Figure S1.** Chromatogram overlay of 2AB labelled N-Glycans released from recombinant (blue) and plamsa-derived (red) Hp variants with PNGase F. Separation was on a Dionex GlycanPac AXH-1, 1.9 μm, 2.1 x 150 mm column using an acetonitrile / 50mM ammonium formate gradient with fluorescence detection. **Table S1.** Peak area by charge-group retention window of 2-AB N-glycans for recombinant human haptoglobin variants and plasma-derived haptoglobin expressed as a percentage of total glycan peak area. (PDF 155 kb)

## Abbreviations

C1r-LP: C1r subcomponent-like protein
Hb: Hemoglobin
Hp: Haptoglobin
HPLC-ESI-MS: High pressure liquid chromatography electrospray ionization mass spectrometry
