# Supplementary material for: Phenotype-specific recombinant haptoglobin polymers co-expressed with C1r-like protein as optimized hemoglobin-binding therapeutics
Source: BMC Biotechnol. 2018 Mar 15;18:15. doi: 10.1186/s12896-018-0424-3 (PMC5856381; doi:10.1186/s12896-018-0424-3)
Supplement: Supplementary file 1 — Figure S1. Chromatogram overlay of 2AB labelled N-Glycans released from recombinant (blue) and plamsa-derived (red) Hp variants with PNGase F. Separation was on a Dionex GlycanPac AXH-1, 1.9 μm, 2.1 x 150 mm column using an acetonitrile / 50mM ammonium formate gradient with fluorescence detection. Table S1. Peak area by charge-group retention window of 2-AB N-glycans for recombinant human haptoglobin variants and plasma-derived haptoglobin expressed as a percentage of total glycan peak area. (PDF 155 kb) [file 12896_2018_424_MOESM1_ESM.pdf]

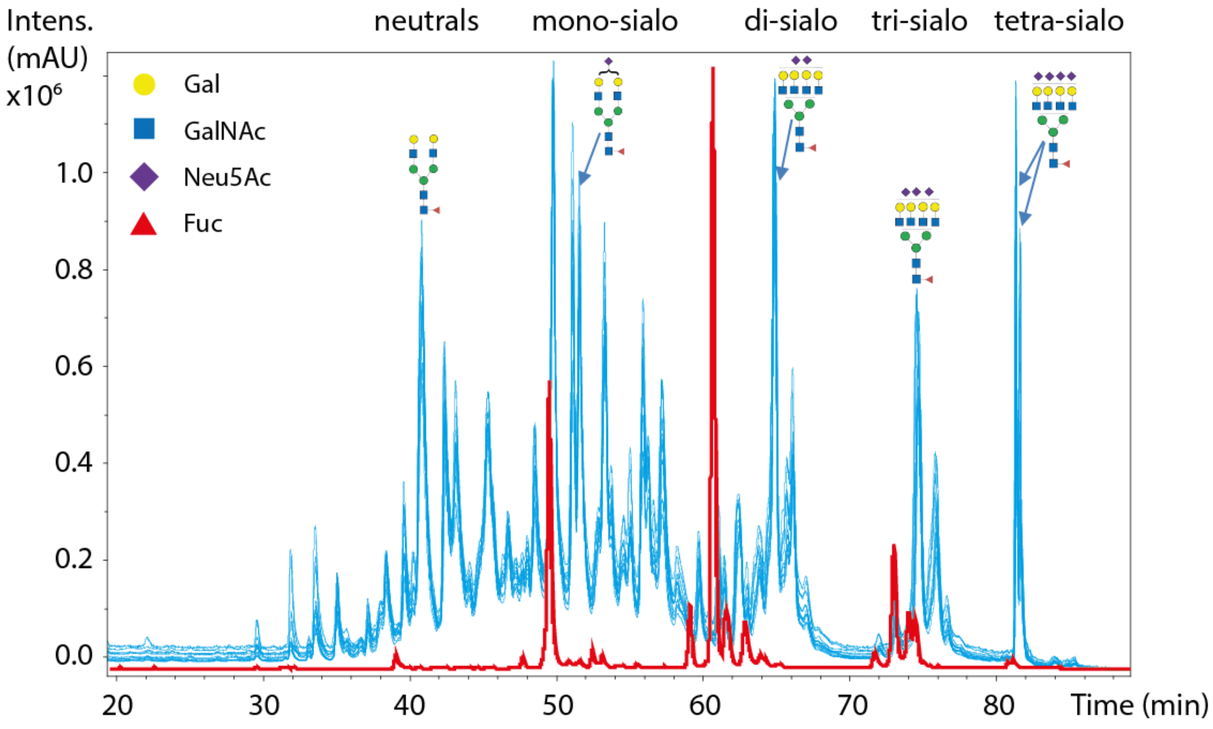

Supplementary Figure 1: Chromatogram overlay of 2AB labelled N-Glycans released from recombinant (blue) and plamsa-derived (red) Hp variants with PNGase F. Separation was on a Dionex GlycanPac AXH-1, 1.9 µm, 2.1 x 150 mm column using an acetonitrile / 50mM ammonium formate gradient with fluorescence detection.

HPLC-ESI-MS analysis of the 2-AB labelled N-glycans from recombinant human haptoglobin variants revealed biantennary complex glycans in a neutral and monosialylated forms and tetraantennary complex glycans in disialylated, trisialylated and tetrasialylated forms. The predominant glycan structures found in the recombinant human haptoglobin variants were core fucosylated. These generalized structural assignments were based on retention times and mass spectra.

Near identical HPLC profiles of different production batches (blue traces) of recombinant Hp1 and Hp2 demonstrate reproducible N-glycosylation. The chromatograms were divided into retention time windows based on glycan anionic charge and the relative peak area of each retention window, which is expressed as a percentage of total area in Supplementary Table 1.

In comparison to recombinant human haptoglobin variants, HPLC-ESI-MS analysis of the 2-AB labelled N-glycans from plasma-derived human haptoglobin (red trace) showed biantennary complex glycans predominantly disialylated, with some monosialylated, and very little neutral glycans. Plasma derived haptoglobin shows proportionally much less trisialylated and tetrasialylated glycans relative to disialylated glycans. Unlike those of the recombinant human haptoglobin variants, the predominant glycan structures found in the plasma derived human haptoglobin were not core fucosylated.

**Sample Hu-Haptoglobin**	**2-AB Glycan % of total peak area by charge group retention window**					
	**neutrals**	**mono-sialo**	**di-sialo**	**tri-sialo**	**tetra-sialo**	**> tetra-sialo**
1F-8His	24.0	39.2	21.3	10.7	4.6	0.2
1F-8His	30.1	38.7	18.6	8.9	3.6	0.2
1S-8His	27.1	39.5	19.6	9.6	4.0	0.2
1S-8His	30.3	39.4	18.4	8.6	3.2	0.1
1S-8His	29.4	39.3	19.0	9.0	3.1	0.2
2FS-8His	27.0	39.4	19.7	9.6	4.1	0.2
2FS-8His	31.6	40.6	17.6	7.6	2.4	0.1
2FS-8His	33.2	39.8	17.0	7.6	2.3	0.1
1S(Q101A,R102A)-8His	24.5	40.9	20.9	9.7	3.8	0.2
1S(Q101A,R102A)-8His	24.0	41.4	21.0	9.6	3.9	0.2
2FS(Q160A,R161A)-8His	28.8	41.9	18.1	8.4	2.6	0.1
2FS(Q160A,R161A)-8His	28.4	43.7	18.1	7.4	2.3	0.1
2FS(Q160A,R161A)-8His	31.6	41.7	17.1	7.2	2.3	0.1
Plasma-derived Hp	1.4	24.3	53.7	19.6	1	

Supplementary Table 1: Peak area by charge-group retention window of 2-AB N-glycans for recombinant human haptoglobin variants and plasma-derived haptoglobin expressed as a percentage of total glycan peak area.
